# Boosting the adsorptive and photocatalytic performance of MIL-101(Fe) against methylene blue dye through a thermal post-synthesis modification

**DOI:** 10.1038/s41598-023-41451-4

**Published:** 2023-09-04

**Authors:** Mehdi Fattahi, Zohreh Niazi, Fatemeh Esmaeili, Ali Akbar Mohammadi, Mahmoud Shams, Binh Nguyen Le

**Affiliations:** 1 Institute of Research and Development, Duy Tan University, Da Nang, Vietnam; 2 School of Engineering & Technology, Duy Tan University, Da Nang, Vietnam; 3 Chemistry Department, Faculty of Science, Ferdowsi University of Mashhad, Mashhad, Iran; 4 Social Determinants of Health Research Center, Mashhad University of Medical Sciences, Mashhad, Iran; 5 Department of Environmental Health Engineering, School of Health, Mashhad University of Medical Sciences, Mashhad, Iran; 6 Department of Environmental Health Engineering, School of Public Health, Neyshabur University of Medical Sciences, Neyshabur, Iran

**Keywords:** Environmental sciences, Chemistry

## Abstract

Photocatalytic degradation under ultra-low powered light is a viable advanced oxidation process technique against extensive emerging contaminants. As a new and remarkable class of nanoporous materials, metal-organic frameworks (MOFs), attract interest for the supreme adsorptive and photocatalytic functionalities. An outstanding MOF, MIL-101(Fe) chosen as a photocatalyst template for the synthesis of α-Fe_2_O_3_ by a simple thermal modification to improve the structural properties toward methylene blue (MB) eradication. Octahedron-like α-Fe_2_O_3_ photocatalyst (Modified MIL-101(Fe), M-MIL-101(Fe)) was superior in dispersion and separation properties in aqueous medium. Moreover, the adsorptive and catalytic performance was increased for modified form by ~ 7.3% and ~ 17.1% compared to pristine MIL-101(Fe), respectively. Synergistic improvement of MB removal achieved by simultaneous adsorption/degradation under 5-W LED irradiation. Parametric study indicated an 18.1% and 44.5% improvement in MB removal was observed by increasing pH from 4 to 10, and M-MIL-101(Fe) dose from 0.2 to 1 g L^−1^, respectively. MB removal followed the pseudo-second-order kinetics model and the process efficiency dropped by 38% as MB concentration increased from 5 to 20 mg L^−1^. Radical trapping tests revealed the significant role of ${\mathrm{OH}}^{.}$ and electron radicals as the major participants in dye degradation. A significant loss in the efficiency of M-MIL-101(Fe) was observed in the reusability tests that is good to study further. In conclusion, a simple thermal post-synthesis modification on MIL-101(Fe) improved its structural, catalytic, and adsorptive properties against MB.

## Introduction

Water resource pollution by emerging organic contaminants poses a worldwide risk to human health and the environment^1–3^. Of organic contaminants, dyes are among the significant sources of water pollution^4^. A huge quantity of dyes is discharged to the environment from a variety of industries such as dye manufacturing, pulp and paper, tannery, textile, plastic, etc. The presence of dyes in aquatic environments could suppress photosynthesis and oxygen availability, increasing the biochemical oxygen demand (BOD), toxicity to human and aquatic flora, and interference with water perspectives^5^.

Of dyes, azo dyes are widely used in different industries such as printing, textiles, leather, and cosmetics due to their premium water solubility and stability^6,7^. Most azo dyes are non-biodegradable, toxic, and carcinogenic so their discharge to the environment must control to abate their negative consequences^8–11^. So far, various treatment methods such as separation, sedimentation, centrifugation, membrane filtration, physical adsorption, and advanced oxidation processes (AOPs) have been used for treating dye streams^12–15^. However AOPs recognized as a promising option as they advantage of a high reaction rate, absence of secondary pollution, economic feasibility, and environmentally benign nature^16^. Photocatalytic degradation is a viable AOP technology that degrades pollutants by reactive radicals i.e. hydroxyl (^.^OH) and superoxide (^.^O_2_^-^) that produce by irradiation of a catalyst^17,18^. Solar-driven and low-powered photocatalysis is a desired technique to abate contaminants due to its sustainability, high efficiency, cost-effectiveness, and feasible operation^19–21^. The photocatalytic activity of a material depends on the ability to produce electron–hole pairs, which in turn produce oxidizing radicals^22^. Semiconductors have been used extensively as photocatalysts to eliminate organic dyes in wastewater^21,23–27^. However, many studied semiconductors suffer from the drawbacks of inadequate efficiency, low surface area, high production cost, fast electron–hole recombination, and toxicity^22^.

As a modern class of hybrid porous nanomaterials, metal–organic frameworks (MOFs), have currently received extensive attention as photocatalysts because of their unique properties such as high surface area and porosity, low toxicity, good chemical stability, facility of synthesis, and tunable composition^20,28–32^. Besides, MOFs are widely investigated for electro/photocatalysis, drug delivery, sensing, gas storage, electronics, and separation^33^.

Of MOFs, Fe-based MOFs exhibited an unexpected light absorption and photocatalytic performance due to their internal Fe–O clusters and good water and light stability during the catalytic process^34^. Fe-based MOFs have also low preparation costs and are environmentally friendly^35^. Particularly, MIL-101(Fe) (MIL, Material Institute Lavoisier) has significant potential in photodegradation thanks to its adjustable internal surface properties, good thermal stability, easy recycling, and favorable electrical properties^17,19^. Jin et al.^36^ prepared MIL-101(Fe)@MIL-100(Fe) for the photodegradation of tetracycline (TC). MIL-101(Fe)@MIL-100(Fe) revealed a promising photocatalytic activity against TC photodegradation compared to MIL-101(Fe) and MIL-100(Fe). Xiao et al.^37^ synthesized cobalt-doped MIL-101(Fe) as an efficient photocatalytic system for the degradation of rhodamine B (RhB). They achieved a > 99% removal of RhB as a model pollutant within 15 min. Song et al.^38^ prepared a nitrogenous core–shell MIL-101(Fe)-based nanocomposite by using a poly-nitrogen conjugated molecule for photodegradation and adsorption of TC. They achieved the removal efficiency and maximum adsorption capacity of 95% and 576.13 mg g^−1^, respectively.

In recent years, MOFs were considered as pristine templates to obtain metal oxide nanoparticles (NPs) with different morphologies. More importantly, MOFs-derived metal oxides usually exhibited high catalytic activity. Among iron oxides, α-Fe_2_O_3_ has been widely used as a photocatalyst because of its non-toxic nature, narrow band gap (2.0–2.2 eV), high efficiency, and economic considerations^39,40^. α-Fe_2_O_3_ is an eco-friendly n-type semiconductor that can absorb light up to 600 nm due to its narrow band gap^41,42^. It is the most chemically stable form of iron oxides under ambient conditions^43^. Studies indicated that the physical and chemical properties of iron oxides are strongly affected by their structural properties. Various techniques such as sol–gel, hydrothermal, microwave/ultrasound, co-precipitation, and combustion have been used to synthesize α-Fe_2_O_3_ NPs with different morphologies. However, expensive substances or complicated equipment are used in most earlier processes^42,44,45^. Interestingly, MOFs-derived metal oxides are viable to generate porous/hollow nanostructures by keeping the previous morphologies during the calcination. For example, Li et al.^46^ synthesized magnetic porous Fe_3_O_4_ /carbon octahedra by two-step calcination of MIL-101(Fe). The porous Fe_3_O_4_/carbon octahedra revealed enhanced catalytic performance for the degradation of methylene blue (MB), as well as good recyclability and stability. Xu et al.^47^ prepared four types of α-Fe_2_O_3-x_ with different morphologies (sphere, octahedron, spindle, and rod) via the pyrolysis of benzimidazole-modified Fe-MOFs precursors as templates. The rod-like α-Fe_2_O_3-x_ exhibited the highest activity for the complete MB degradation with an apparent reaction rate constant k = 0.08 min^−1^. Zan et al.^48^ synthesized a series of Fe_2_O_3_ nanomaterials by pyrolysis of MIL-53(Fe) at different temperatures. They found that Fe_2_O_3_ synthesized using MIL-53 (Fe) at 500 °C has a better catalytic performance than commercial Fe_2_O_3_ and Fe_2_O_3_ prepared directly by FeCI_2_·6H_2_O for degrading RhB.

In this study, a nonporous structure, octahedron-like α-Fe_2_O_3_ (Modified MIL-101(Fe), M-MIL-101(Fe)) with improved photocatalytic and adsorptive performance was developed by simple thermal modification of MIL-101(Fe). MB which belongs to azo dyes was used as a common cationic model pollutant to study the adsorptive and catalytic performance of M-MIL-101(Fe). The photocatalytic experiments were accomplished under the ultra-low-power 5-W LED lights and promising removal efficiency obtained by a viable light irradiation source for M-MIL-101(Fe). The study covers parametric examination of operating variables, kinetic modeling, radical trapping experiments, and reusability tests. Also, the structural robustness of M-MIL-101(Fe) after several application cycles was examined by different characterization techniques.

## Materials and methods

### Chemicals

The entire chemicals used for the synthesis of MIL-101(Fe) (terephthalic acid (TPA), ferric chloride hexahydrate (FeCl_3._6H_2_O), dimethylformamide (DMF), and ethanol (ETOH)), and the experiments including MB, sodium hydroxide (NaOH), hydrochloric acid (HCl), silver nitrate (AgNO_3_), isopropanol (IPA), ascorbic acid (AA), and potassium iodide (KI) were pure and AR grade. Deionized water (DIW) was used to prepare dye solutions.

### Instruments

The UV–vis diffuse reflectance spectroscopy (UV–vis DRS) was carried out in a DRS S_4100 SCINCO spectrophotometer. The photoluminescence (PL) spectra of MIL-101(Fe) and M-MIL-101(Fe) were recorded using Perkinelmer LS 45 spectrometer at room temperature. The FTIR spectra of MIL-101(Fe) and M-MIL-101(Fe) were evaluated in the range of 400–4000 cm^−1^ by a KBr pellet on a Thermo Nicolet-Avatar 370 Spectrometer at room temperature. The X-ray diffraction (XRD) patterns of the synthesized materials were recorded by a Bruker/D8 Advanced diffractometer by using a CuKα (λ = 0.15406 nm) radiation. The surface morphology and energy-dispersive X-ray spectroscopy (EDS) of prepared materials were investigated through the field-emission scanning electron microscopy (FE-SEM, MIRA3 TESCAN, Czech Republic).

### Preparation of MIL-101(Fe)

Fe-based MOF, MIL-101(Fe), was prepared based on the previously protocol in DMF as solvent and TPA as organic linker for Fe ions^46,49^. 0.412 g of TPA and then 1.3244 g of FeCl_3._6H_2_O were dissolved in DMF (30 mL) followed by sonication for 10 min. The clear solution was then transferred into a Teflon-lined stainless-steel autoclave and heated at 110 °C for 20 h. After crystallization period and when the autoclave cooled to ambient temperature, the orange precipitates separated centrifugally and washed frequently by ETOH and fresh DMF. The crystals dried in an oven at 60 °C overnight.

### Preparation of modified MIL-101(Fe)

**Figure 1:**
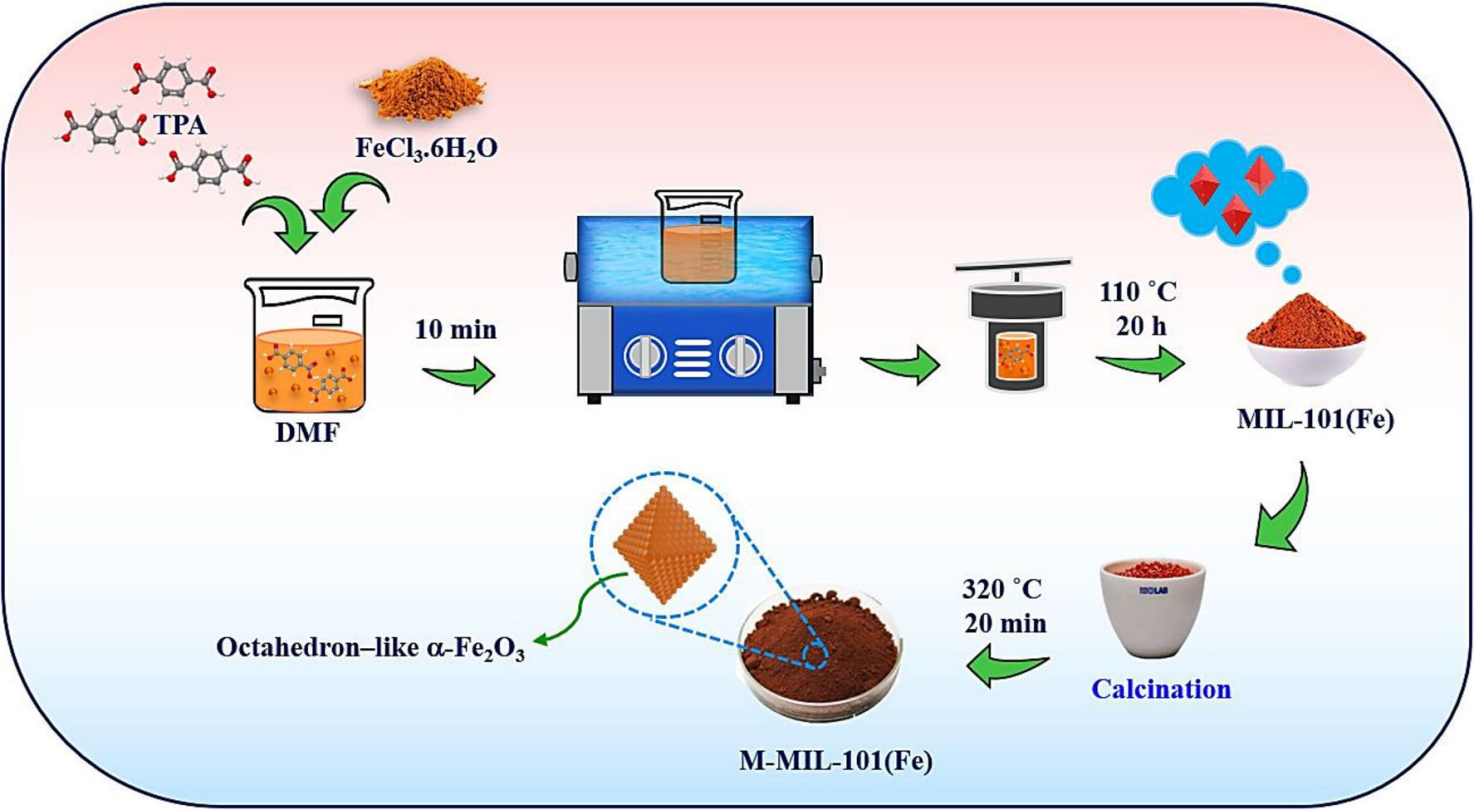
Schematic illustration for the preparation of MIL-101(Fe) and M-MIL-101(Fe).

The orange MIL-101(Fe) powder was transferred to an open cruiser where heated at 320 °C for 20 min. The orange powder turns brown after the calcination in air. The schematic illustration for the preparation of MIL-101(Fe) and M-MIL-101(Fe) is shown in Fig. 1.

### Adsorption/photocatalytic studies

Adsorption and photocatalytic degradation of prepared MIL-101(Fe) and M-MIL-101(Fe) were determined in batch mode system using MB as the model contaminant. The experimental study consists of preliminary tests to evaluate the removal efficiency of pristine MIL-101(Fe) and M- MIL-101(Fe) against MB. The adsorptive properties and photocatalytic degradation performance of the materials were assessed by conducting the process under the dark and light irradiation, respectively.

A simple LED module was used to supply light using 5-W LED lamps. Using LED module have the advantage of energy-efficient, durable, low-cost, and available source of light for lab studies and real treatment units.

Once determined which MIL-101(Fe) or M-MIL-101(Fe) was more efficient in MB removal, the study continued to assess the effect of environmental condition on the process. One factor at a time (OFAT) method was opted to determine the significance of pH, contact time, catalyst dose, and MB concentration on the process. The levels of studied variables in photocatalytic degradation of the dye are presented in Table 1.

**Table 1:** Factors and their levels studied in MB degradation by M-MIL-101(Fe).

Factor	Unit	Study levels			
pH	–	4	6	8	10
Contact time	min	15	30	60	90
Catalyst dose	g L^−1^	0.1	0.25	0.5	0.75
MB concentration	mg L^−1^	5	10	15	20

The degradation efficiency of MB was calculated using Eq. (1) by the difference between the initial ($C_{0}$) and final concentration ($C$) as measured by spectrophotometer at 665 nm:

1 $$Degradation \;efficiency \% \; = \;\frac{{\left( {C_{0} - C} \right)}}{{C_{0} }}\; \times \;100$$

### Scavenger tests

Scavenger tests are useful to identify the role of radicals in pollutant degradation, and to propose the mechanism of pollutant destruction. In scavenger test, a chemical agent that block specific radical added to the photocatalytic system and the system efficiency then analyzes for significance of active species. In this study, AgNO_3,_ IPA, AA, and KI with a concentration of 2 mmol L^−1^ used to block electron (${\text{e}}^{ - }$), hydroxyl radical (${\text{OH}}^{ \cdot }$), superoxide radical (${\text{O}}_{2}^{ \cdot - }$), and hole (${\text{h}}^{ + }$), respectively. Additional experiments for the reusability of M-MIL-101(Fe), kinetic of MB removal, and structural stability of photocatalyst accomplished that discussed in detail in the following sections.

## Results and discussion

### Characterization

**Figure 2:**
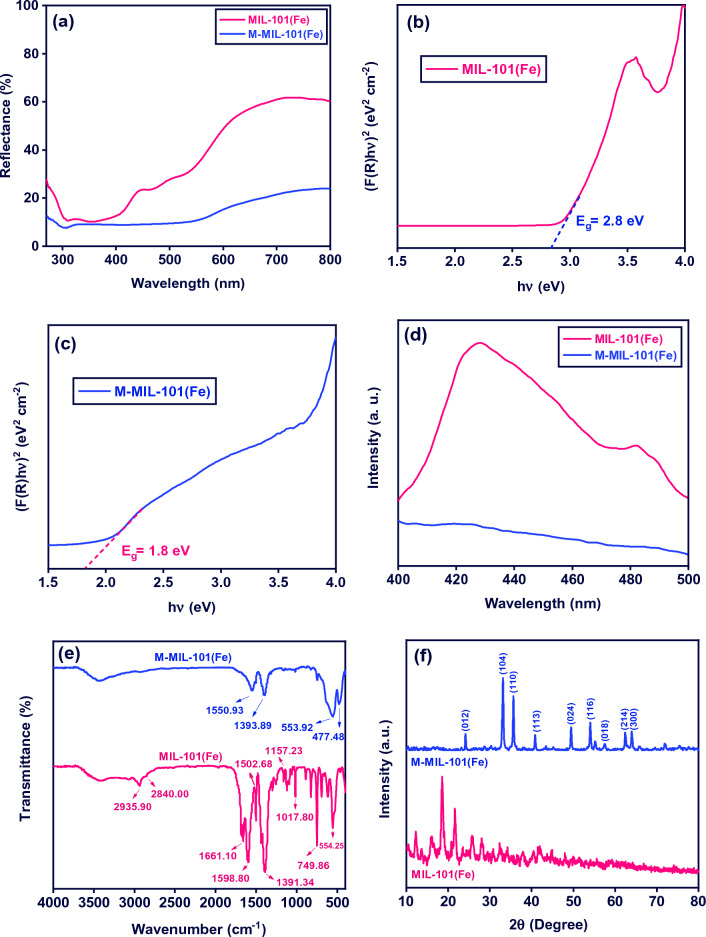
(**a**) The UV–vis DRS, (**b**, **c**) Kubelka–Munk transformed reflectance spectra, (**d**) PL spectra, (**e**) FT-IR spectra and (**f**) XRD patterns of MIL-101(Fe) and M-MIL-101(Fe).

The optical properties of the MIL-101(Fe) and M-MIL-101(Fe) were examined by UV–vis DRS (Fig. 2a). The MIL-101(Fe) shows high light reflectance in the range of 550–800 nm. For M-MIL-101(Fe), the light reflectance strongly decreased in this range, indicating a high visible light absorption. The band gap of samples was calculated by the Kubelka–Munk equation. From the DRS, the Kubelka–Munk function is proportional to the extinction coefficient (α) and R is the reflectance^50^:

2 $$\alpha = F\left( R \right) = \frac{{\left( {1 - R} \right)^{2} }}{2R}$$

Then, the band gap energies of the samples were calculated by Eq. (3):

3 $$\left( {\alpha h\nu } \right)^{n} = A \left( {h\nu - E_{g} } \right)$$

where hν, A, and E_g_ are the energy, proportionality constant, and band gap energy, respectively. In this equation, n can be 2 or ½ for direct and indirect semiconductors, respectively.

As illustrated in Fig. 2b, MIL-101(Fe) shows the band gap of 2.8 eV, while M-MIL-101(Fe) (Fig. 2c) shows lower bandgap of 1.8 eV, which is beneficial for improvements of photocatalytic performances.

It is well documented that the recombination of photogenerated electron–hole pairs reduced the photocatalytic activity as it prevents e^−^/h^+^ to involve in redox reactions to form active radicals^16^. A lower PL emission intensity reflects the less recombination rate of the charge carriers on the surface^17^. The PL spectra of MIL-101(Fe) and M-MIL-101(Fe) are presented in Fig. 2d. As shown, M-MIL-101(Fe) exhibits a lower PL peak intensity compared to MIL-101(Fe), indicating the stronger separation rate of the electron–hole pairs^38^.

The FTIR analysis in the range of 400–4000 cm^−1^ was applied to determine the functional groups for MIL-101(Fe) and M-MIL-101(Fe) as shown in Fig. 2e. The broad vibrational band at about 3200–3700 cm^-1^ for both samples corresponds to the stretching mode of hydroxyl group^51,52^. In the spectrum of MIL-101(Fe), the absorption bands at 2935.90 and 2840.00 cm^−1^ are correspond to the C–H asymmetric and symmetric stretching vibrations, respectively. The band at 1661.10 cm^−1^ arises from the vibration of C=O bond. The bands at 1391.34 and 1598.80 cm^−1^ corresponds to the symmetrical and asymmetrical vibrations of the carboxylic group (–COO–), respectively. The bands at 749.86, 1017.80, and 1157.23 cm^−1^ are assigned to the C–H bending vibration, while the band at 1502.68 cm^−1^ showed the presence of C=C vibration in the benzene ring. The band at 554.25 cm^−1^ related to the Fe–O stretching vibration^36,53^. In M-MIL-101(Fe) spectrum, the bands at 553.92 and 477.48 cm^-1^ are assigned to the stretching vibration of Fe–O in α-Fe_2_O_3_^54^. The week bands at 1393.89 and 1550.93 cm^−1^ can be attributed to presence of a portion of MIL-101(Fe) in α-Fe_2_O_3_.

The crystalline structure and phase composition of MIL-101(Fe) and M-MIL-101(Fe) were studied by XRD, as shown in Fig. 2f. The XRD pattern of MIL-101(Fe) showed well-defined diffraction peaks at about of 12.2°, 16.1°, 18.8°, and 21.7°, which was in good agreement with those reported in previous literature^55^. All diffraction peaks at about 24.1°, 33.1°, 35.6°, 40.8°, 49.5°, 54.1°, 57.5°, 62.4°, and 64.0° that correspond to the reflection planes of (012), (104), (110), (113), (024), (116), (018), (214), and (300) can be well-indexed to the M-MIL-101 (Fe)^48^.

**Figure 3:**
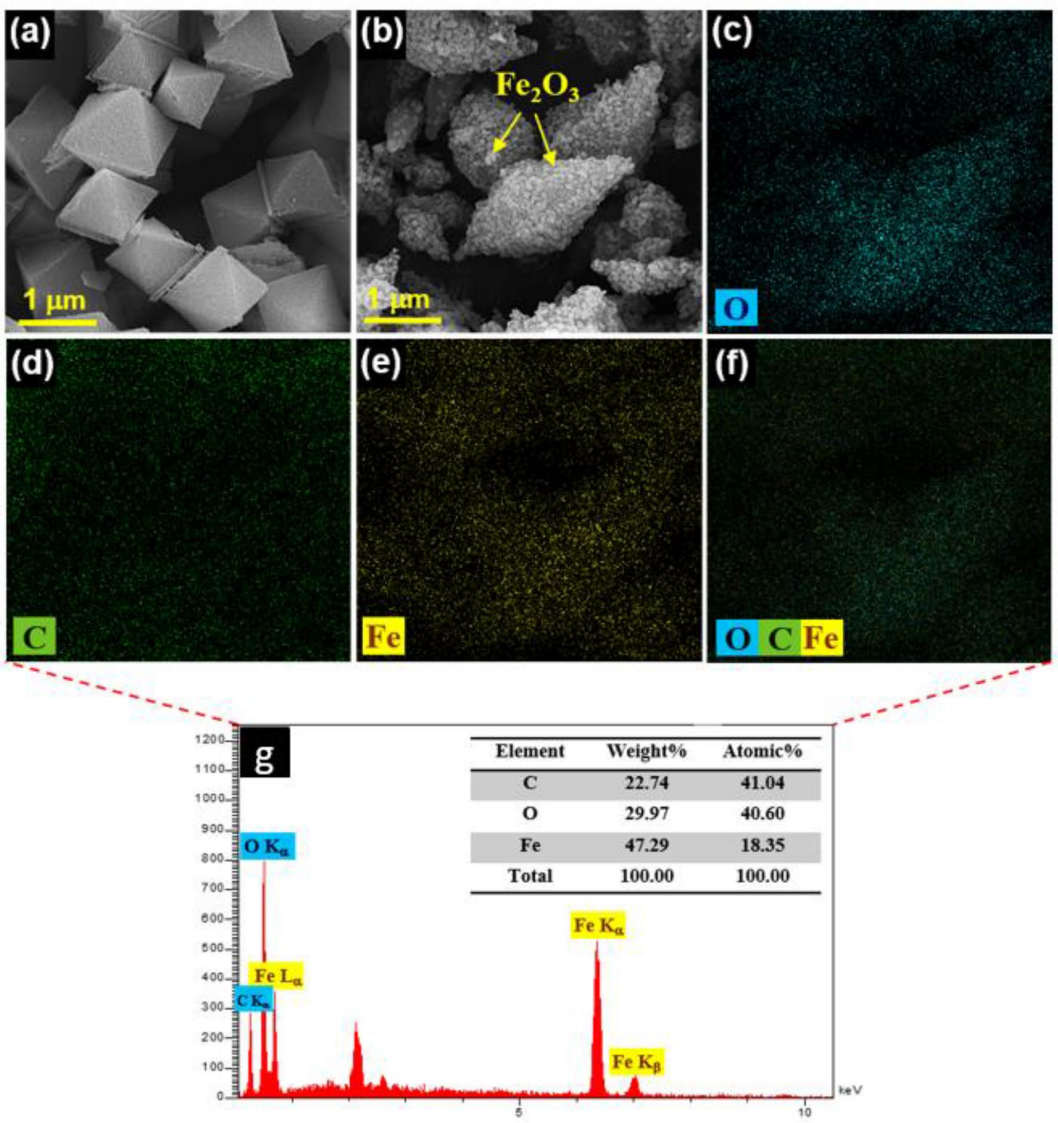
The FE-SEM images of the (**a**) MIL-101(Fe) and (**b**) M-MIL-101(Fe), (**c**–**f**) elemental mapping of O, C, and Fe, (**g**) EDX spectrum for M-MIL-101(Fe).

The surface morphology of MIL-101(Fe) and M-MIL-101(Fe) was investigated by FE-SEM and shown in Fig. 3a and b, respectively. As shown in Fig. 3a, the prepared MIL-101(Fe) exhibited an octahedral structure with a smooth surface and clear edges. After calcination in air, the octahedron-like α-Fe_2_O_3_ was successfully synthesized. Furthermore, the elemental mapping shows an almost uniform distribution of elements on M-MIL-101(Fe) surface in Fig. 3c–f. The EDX spectrum of M-MIL-101(Fe) (Fig. 3g) confirmed the presence of C, O, and Fe.

### MIL-101(Fe) and M-MIL-101(Fe) removal efficiency

**Figure 4:**
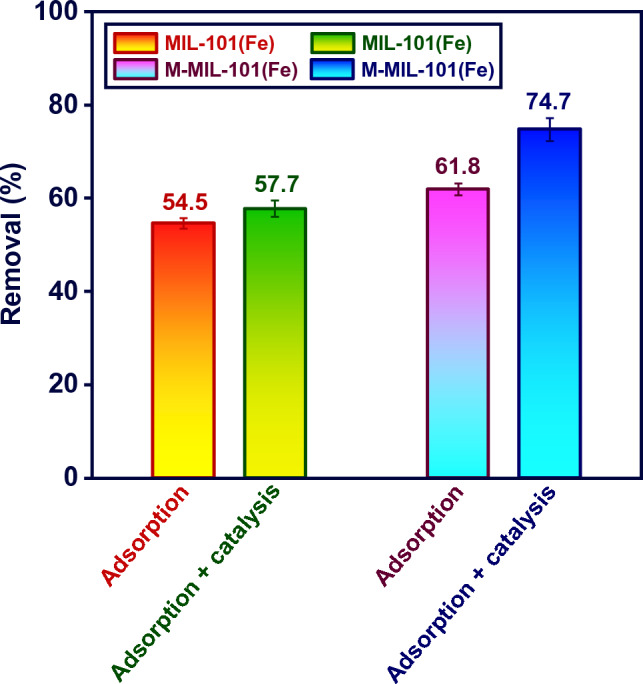
Photoassysted and adsorptive removal of MB by MIL-101(Fe) and M-MIL-101(Fe) (MB: 10 mg L^−1^, dose: 0.5 g L^−1^, reaction time: 90 min).

To compare the dye elimination efficiency, adsorption and photo assisted catalytic degradation of MB were performed for the synthesized materials. A fixed dose (0.5 g L^−1^) of MIL-101(Fe) or M-MIL-101(Fe) added to the dye solutions (10 mg L^−1^) while the process completed for 90 min under LED light irradiation (catalytic degradation) or in the dark environment (adsorption). The difference between the adsorption + catalysis removal columns for the materials in Fig. 4 reflected the catalytic degradation of M-MIL-101(Fe) that is almost negligible for MIL-101(Fe). As seen, M-MIL-101(Fe) exhibited a superior adsorptive and catalytic performance against MB compared to pristine MIL-101(Fe). The adsorptive and catalytic removal efficiencies for M-MIL-101(Fe) were ~ 7.3% and 17.1% more than MIL-101(Fe), respectively. In addition to a supreme removal property, M-MIL-101(Fe) takes advantage of a good dispersibility and separability in water medium. Interestingly, M-MIL-101(Fe) could easily separate by magnetic field. Moreover, M-MIL-101(Fe) separate easier to produce a clear solution by centrifugation as it is denser than MIL-101(Fe).

### Influence of operating parameters on MB degradation

**Figure 5:**
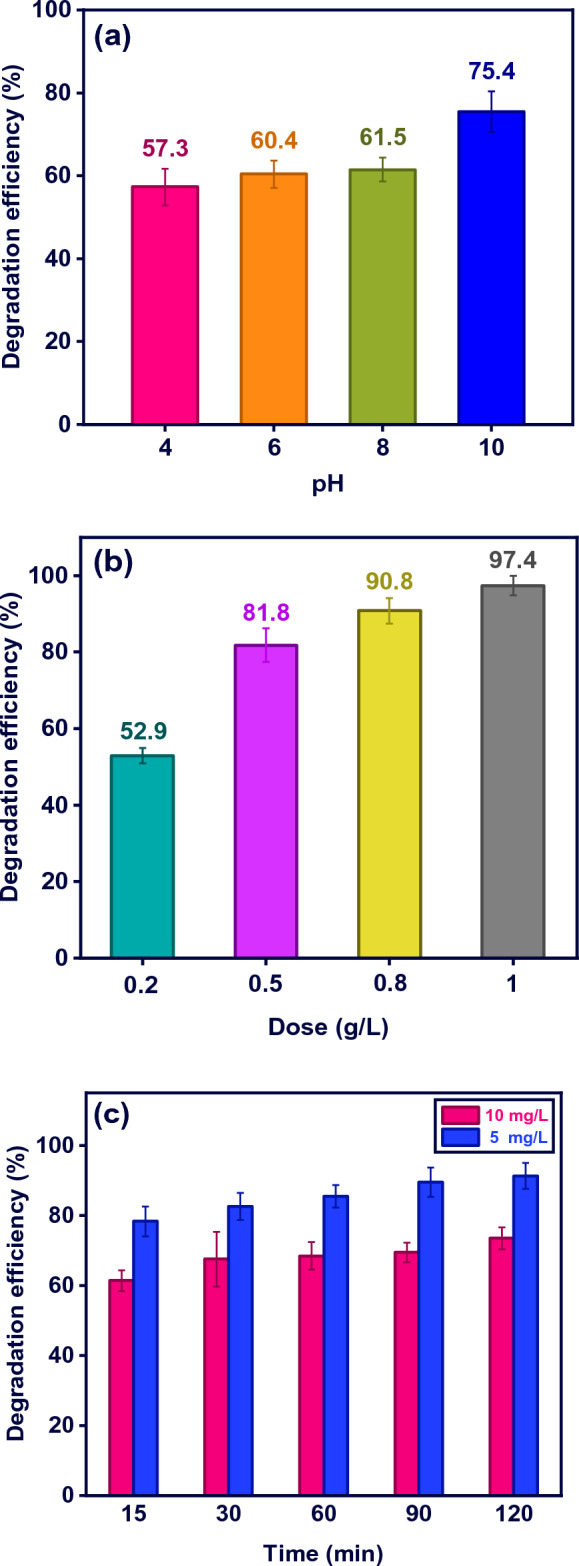
Photoassysted removal of MB by M-MIL-101(Fe) as a function of (**a**) pH, (**b**) M-MIL 101 dose, and (**c**) MB concentration.

To evaluate the photocatalytic performance of M-MIL-101(Fe), various parameters such as pH, catalyst dosage, and initial dye concentration were investigated. The initial solution pH is an important factor in the physicochemical properties of materials and solutes. The effect of solution pH in photocatalytic performance of M-MIL-101(Fe) was studied at different pH i.e. 4, 6, 8, and 10. As shown in Fig. 5a, increasing the solution pH from 4 to 10, led to an improvement in the photodegradation degradation of MB from 57.2 to 75.3%.

The effect of catalyst dose on the dye degradation was also studied in the range of 0.2–1 g L^−1^ and the results are shown in Fig. 5b. The photodegradation efficiency of MB improved significantly from 52.9 to 97.4% with an increase in the photocatalyst dosage from 0.2 to 1 g L^−1^. This could be attributed to the increase in active sites for MB adsorption and also generating more active species during the irradiation. A dose of 0.5 g L^−1^ was chosen for the following experimental steps.

The pollutant concentration also is a significant parameter that affects process efficiency. Thus, MB removal in the concentrations of 5 and 10 mg L^−1^ were investigated. Figure 5c indicated the degradation efficincy was decreased by the dye concentration. A lower light penetration and decrease in the energy of photons reach the photocatalyst surface, leading to a decrease in the generated oxidizing radicals is a possible cause of drop in MB removal at the elevated concentrations.

### Kinetic study

The kinetic of dye degradation was examined by two kinetic models i.e. the pseudo-first order and pseudo-second order. The non-linear forms of pseudo-first order and pseudo-second order kinetic models are given in Eqs. (4) and (5), respectively^56^:

4 $$C = C_{0} \times \exp \left( { - k_{1} t} \right)$$

5 $$C = \frac{{C_{0} }}{{1 + C_{0} k_{2} t}}$$

where *k*_*1*_ and *k*_*2*_ are the rate constants for pseudo-first order and the pseudo-second order, respectively. *C*_*0*_, *C*, and *t* are the initial concentration of MB, the concentration of MB at the time t, and reaction time, respectively.

**Figure 6:**
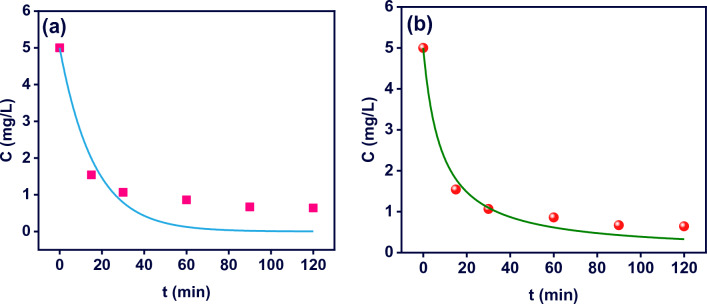
(**a**) Pseudo-first order and (**b**) pseudo-second order kinetic models for MB degradation by M-MIL-101(Fe) (pH 10, MB concentration: 5 mg L^−1^, and M-MIL-101(Fe) dose: 0.5 g L^−1^).

Figures 6a and b shows the pseudo-first order and pseudo-second order models fitted on MB degradation data. The kinetic constants of *k*_*1*_ and *k*_*2*_ and statistical parameters i.e., the coefficient of determination (*R*^*2*^), adjusted R-square $\left( {R_{adj}^{2} } \right)$, and the residual sum of square (*RSS*) for MB degradation by M-MIL-101(Fe) are outlined in Table 2. From the Table 2, it is clearly seen that the *R*^*2*^ and ${R}_{adj}^{2}$ values for pseudo-second order model are higher than the pseudo-first order model that indicate the degradation of MB obeys the pseudo-second order model. Furthermore, the smaller value of *RSS* for the pseudo-second order model than the other model confirms the process obeys this kinetic model.

**Table 2:** The kinetic constants and statistical parameters for MB degradation.

Kinetic model	Parameters	MB concentration
		5 (mg L^−1^)
Pseudo-first order	${k}_{1}$ (min^−1^)	0.06 ± 0.01
	${R}^{2}$	0.88
	${R}_{adj}^{2}$	0.88
	$\mathrm{RSS}$	1.65
Pseudo-second order	${k}_{2}$ (L mg^−1^ min^−1^)	0.02 ± 0.00
	${R}^{2}$	0.98
	${R}_{adj}^{2}$	0.98
	$\mathrm{RSS}$	0.28

### Radical trapping tests

**Figure 7:**
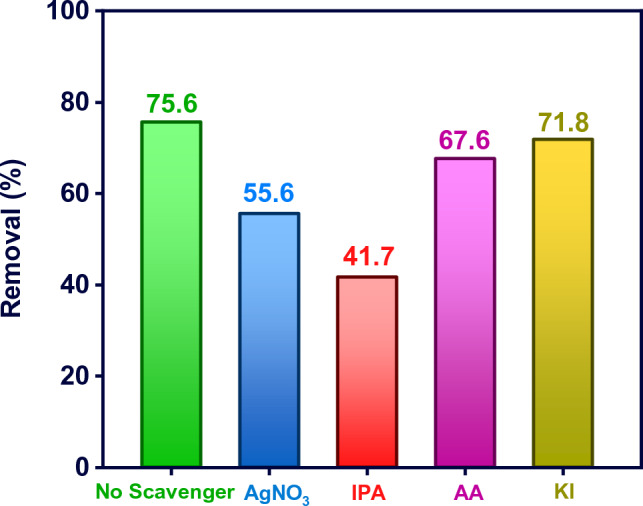
The effect of 2 mmol L^−1^ radical scavengers on the photocatalytic degradation of MB by M-MIL-101(Fe) (MB concentration: 10 mg L^−1^, dose: 0.5 g L^−1^, reaction time: 90 min).

To determine the mechanism of MB photocatalytic degradation using M-MIL-101(Fe), radical trapping of major active oxidation species was performed. AgNO_3_, IPA, AA, and KI were applied as the scavengers of electron (${\text{e}}^{ - }$), hydroxyl radical (${\text{OH}}^{ \cdot }$), superoxide radical (${\text{O}}_{2}^{ \cdot - }$), and hole (${\text{h}}^{ + }$), respectively. As shown in Fig. 7, the MB removal efficiency was significantly reduced to 55.6 and 41.7% by adding AgNO_3_ and IPA into the reaction system, respectively. These results reveal that ${\text{OH}}^{ \cdot }$ and electron radicals play a significant role in the photocatalytic degradation of MB. However, the AA and KI have a negligible effect on the removal efficiency, indicating that ${\text{O}}_{2}^{ \cdot - }$ and ${\text{h}}^{ + }$ are not the main active species for the photocatalytic degradation of MB.

### MB removal from synthetic wastewater

**Figure 8:**
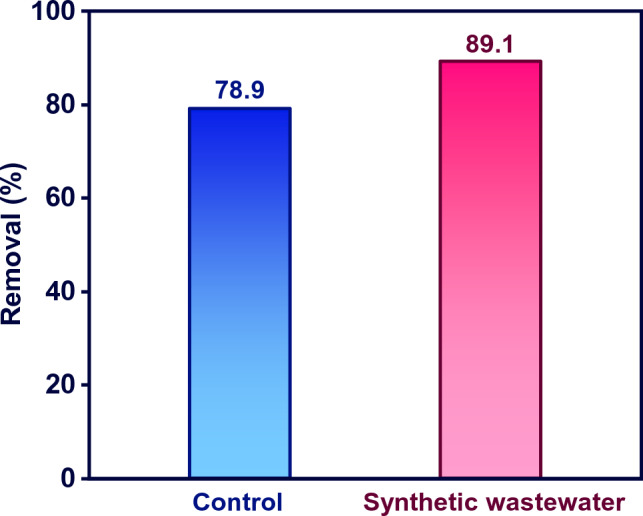
The MB removal percentage for control and real samples (MB concentration: 10 mg L^−1^, M-MIL-101(Fe) dose: 0.5 g L^−1^, and reaction time: 90 min).

To study the dye removal in real condition in the presence of co-existing ions, dye degradation tests accomplished in a water spiked with MB. The characteristics of water matrix are presented in Table S1. Figure 8 compares MB removal percentage for dye solutions prepared by DIW (control) with those in real samples. Interestingly, the presence of co-existing species improved the removal efficacy and MB removal increased by 10.2% in real sample compared to the solutions prepared by DIW. The presence of coexisting ions in synthetic and real wastewater can significantly affect the photocatalytic removal of contaminants. The type and concentration of coexisting ions, as well as the type of catalyst, can influence the photocatalytic activity. Some earlier reports indicated an improvement in photocatalytic performance in the presence of coexisting ions. For instance, Gao et al.^57^ indicated the photocatalytic degradation of carbamazepine improved in the presence of Ca^2+^, and Mg^2+^. However, HCO^3−^, Cl^−^, and NO_3_^−^ suppressed the removal due to the quenching effects they have on ${\mathrm{OH}}^{.}$ and ${\mathrm{h}}^{+}$. In another study, SO_4_^2-^ showed the highest inhibiting impact on MB removal, while chloride ions increased the degradation of MB by CuO–Cu_2_O nanocomposite^58^.

### Reusability study

**Figure 9:**
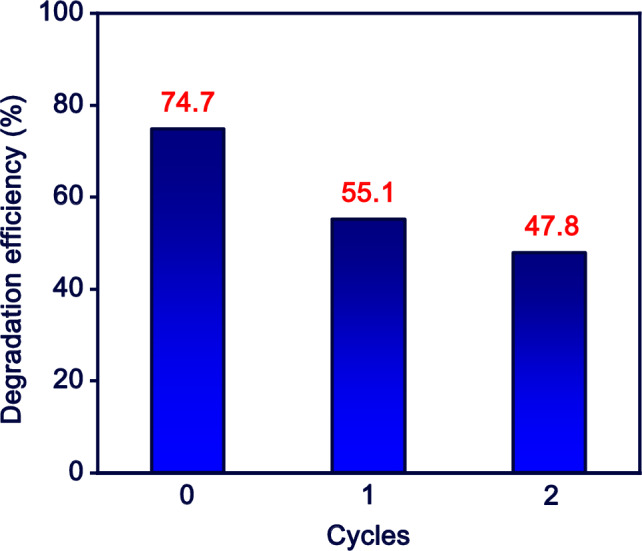
The reusability of M-MIL-101(Fe) in photocatalytic degradation of MB (pH 10, M-MIL-101(Fe) dose: 0.5 g L^−1^, MB concentration: 10 mg L^−1^, and irradiation time: 90 min).

The stability and reusability of photocatalyst play a significant role in the operation of real treatment units. The recyclability of M-MIL-101(Fe) for photocatalytic degradation of MB was evaluated in two successive cycles as shown in Fig. 9. For that, the M-MIL-101(Fe) photocatalyst for each cycle was reused for the next cycle after washing with DIW and dring at 70 °C for 8 h. According to Fig. 9, the degradation efficiency of MB decreased from 74.7 to 47.8% after two cycles. The significant loss in the MB removal efficacy is a disadvantage property for M-MIL-101(Fe) that should alleviate in the future studies.

### Stability

**Figure 10:**
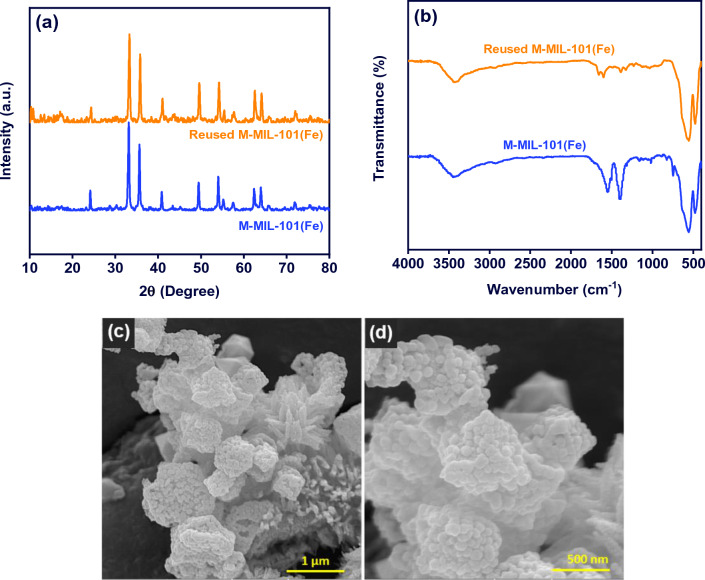
(**a**) XRD patterns, (**b**) FT-IR spectra, (**c**, **d**) FE-SEM images of the exhausted M-MIL-101(Fe) with two magnifications.

To study the stability of M-MIL-101(Fe), the exhausted M-MIL-101(Fe) after two photocatalytic cycles undergoes XRD, FT-IR, and FE-SEM analysis. As shown in Fig. 10a, there is no obvious difference in XRD patterns of M-MIL-101(Fe) and reused M-MIL-101(Fe). Moreover, as shown in Fig. 10b, a same pattern in FT-IR spectra recorded for fresh and exhausted material. In addition, the FE-SEM images of exhausted M-MIL-101(Fe) with different magnifications are shown in Fig. 10c and d. As can be seen, the physical morphology of some crystals affected by consecutive use-reuse cycles. Overally, the characteristic study of the exhausted M-MIL-101(Fe) indicated that in spite of some physical changes in morphology, the crystalline structure and surface functional groups have no significant changes during the photocatalytic process, confirming the good stability and reusability of the photocatalyst.

## Conclusion

AOPs are promising techniques for non-selective abatement of pollutants and attract attentions against emerging contaminants such as dyes. Photocatalytic degradation is an AOP technique with economical and practical advantages, especially when the process operated under low powered light. A particular member of metal organic frameworks, MIL-101(Fe), simply treated by thermal modification to prepare M-MIL-101(Fe) with superior adsorption/catalytic performance. Methylene blue (MB) removal experimented under the irradiation by 5-W LED lamps. Parametric studies indicated that MB removal increased by solution pH and M-MIL-101(Fe) dose, and inversely decreased by MB. ${\text{OH}}^{ \cdot }$ and electron radicals play crucial role in the degradation of MB that obey the pseudo-second order kinetics model. Despite some physical deformation in consecutive use-reuse cycles, the crystallinity and functional groups of M-MIL-101(Fe) still remained intact. Further investigation would be advantageous to improve the reusability of M-MIL-101(Fe).

### Supplementary Information

Supplementary Table S1.

## Data Availability

The datasets generated and analyzed during the current study were available from the corresponding author on reasonable request.
