# Supplementary material for: Boosting the adsorptive and photocatalytic performance of MIL-101(Fe) against methylene blue dye through a thermal post-synthesis modification
Source: Sci Rep. 2023 Sep 4;13:14502. doi: 10.1038/s41598-023-41451-4 (PMC10477185; doi:10.1038/s41598-023-41451-4)
Supplement: Supplementary file 1 — Supplementary Table S1. [file 41598_2023_41451_MOESM1_ESM.docx]

*Supplementary Information*

**Table S1.** The physicochemical properties of synthetic wastewater.

| Value | Unit | Parameters |
| --- | --- | --- |
| 20.5 | ^0^C | Temperature |
| 7.5 | - | pH |
| 36 | mg/L CaCO_3_ | Ca |
| 16.8 | mg/L CaCO_3_ | Mg |
| 11.5 | mg/L | Na |
| 0.11 | mg/L | K |
| 19.2 | mg/L | SO_4_ |
| 7.1 | mg/L | Cl^-^ |
| 0.1 | mg/L | F^-^ |
| 1.5 | mg/L | NO_3_^-^ |
| 145 | mg/L CaCO_3_ | Total Hardness |
| 10 | mg/L | Humic acid |
